# Sclerosing Mesenteritis: Diverse clinical presentations and dissimilar treatment options. A case series and review of the literature

**DOI:** 10.1186/1755-7682-4-17

**Published:** 2011-06-02

**Authors:** Konstantinos Vlachos, Fotis Archontovasilis, Evangelos Falidas, Stavros Mathioulakis, Stefanos Konstandoudakis, Constantinos Villias

**Affiliations:** 1 First Department of General Surgery, 417 NIMTS, Veterans Hospital of Athens, 10-12 Monis Petraki, 11521, Athens, Greece; 2 First Department of Therapeutic Endoscopy and Laparoscopic Surgery, Iaso General Hospital, 264 Mesogion Avenue, 15562, Cholargos, Greece; 3 Department of Pathology,417 NIMTS, Veterans Hospital of Athens,10-12 Monis Petraki, 11521, Athens, Greece

## Abstract

Sclerosing mesenteritis (SM) is a rare pathological condition affecting the mesentery. It is a benign, non-specific inflammation of the adipose tissue of the mesentery of the small intestine and colon. It is characterized by a variable amount of chronic fibrosis. Its etiology is unknown, the pathogenesis is obscure, while the pathological characteristics of the disease are unspecific. The initial clinical presentation varies from typically asymptomatic to that of an acute abdomen. The diagnosis is suggested by computed tomography but is usually confirmed by surgical biopsies. Treatment is largely empirical; it is decided upon on the basis of the clinical condition of the patient, and usually a few specific drugs are used. Surgical resection is sometimes attempted for definitive therapy, although the surgical approach is often limited. We will present five cases of SM as well as a review of the available literature in order to state and compare a variety of clinical presentations, diverse possible etiologies and dissimilar treatment options.

## Introduction

Sclerosing mesenteritis (SM) is a rare, non-specific, benign and chronic fibroinflammatory disorder of unknown etiology that primarily affects the small bowel mesentery. It usually involves the root of the small bowel mesentery, but it can also involve the mesocolon, the peripancreatic and omental fat, and infrequently the retroperitoneal or pelvic fat [1-6]. The first known series was published in 1924 [2,7]. In that series the disease was described under the names of "retractile mesenteritis" and "mesenteric sclerosis". Since then these terms have undergone serious elaboration on the basis of the predominant histology in order to better describe the disease, including mesenteric lipodystrophy (predominantly fatty degeneration and necrosis), mesenteric panniculitis (marked chronic inflammation) and retractile mesenteritis or mesenteric fibrosis (predominant fibrosis) [4,6]. Furthermore, mesenteric Weber-Christian disease, liposclerotic mesenteritis, lipomatosis and lipogranuloma of the mesentery are numerous other names that have been used to describe the disease [8]. Emory et al in 1997 [1], after a thorough review of 84 cases concluded that all this uncertainty is partly due to the variable histological features of a single pathological entity characterized by a non-specific inflammatory processes in the mesenteric fat, that may ultimately lead to retraction and fibrosis. This varied terminology has caused considerable confusion, but the condition can now be evaluated as a single disease with two pathological subgroups. When inflammation and fatty necrosis are predominant components of the process, it is called "mesenteric panniculitis", while if fibrosis and retraction are the principals components, it is called ''retractile mesenteritis''. The overall presence of some degree of fibrosis makes the pathological term "sclerosing mesenteritis" the most accurate and preferred term in most cases [1]. More than 250 cases have been reported in world literature [9-12] and are often diagnosed incidentally, while SM shows a 0, 6% prevalence in patients undergoing abdominal computed tomography (CT) for various reasons [13]. The rarity of this condition has restricted the ability to record demographic and clinical features, natural history and response of the disease to therapy. Thus, treatment decisions are guided by anecdotal/empirical experience and small case studies. Various drugs, including corticosteroids, immunosuppressives, colchicine, tamoxifen, progesterone, and recently thalidomide have been used with varying success [14-17].

### Case 1

**Figure 1:**
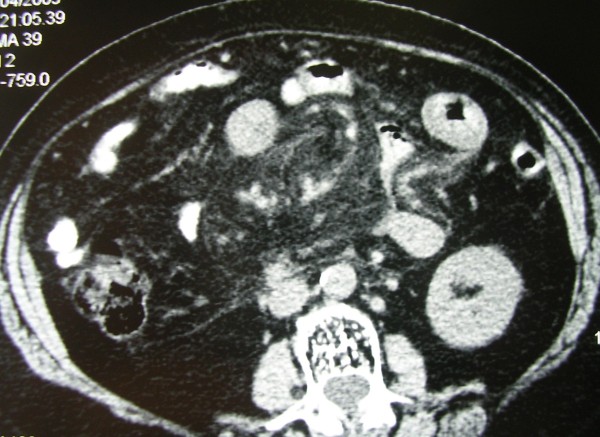
**Axial contrast enhanced CT demonstrates a well marginated fat attenuation of the mesentery surrounding the mesenteric vessels**. A halo of fat is preserved around the mesenteric vessels and nodules. The lesion is closely related to the adjacent opacified small bowel which is peripherally displaced. The fatty mass is delineated by a hyperdense stripe and accompanied by multiple small nodules.

An 82-year-old female patient was admitted to our hospital with a six month history of recurrent left-sided abdominal pain, mainly located at the left hypochondrium, bloating and nausea. Her past medical history included rheumatoid arthritis under prednisolon and methotrexate. These medications had been ceased 9 months earlier due to anemia. Since then she had not taken any drugs. She had no known allergies, no significant family history and a review of her systems was unremarkable. Upon physical examination the patient had diffuse abdominal pain in the left abdomen. Deep palpation of the abdomen revealed an ill-defined mass located at the left upper abdominal quadrant. Her laboratory profile was normal. Abdominal x-ray did not reveal any signs of ileus. Abdominal computed tomography (CT) was performed after oral and intravenous contrast administration, which showed a focal increase in density of the mesenteric fat with stranding in the supra-umbilical and left hypochondrium regions, which was most probably inflammatory in origin and suggestive of mesenteric panniculitis (Figure 1). Diverticulosis of the sigmoid colon was noted as well. The patient underwent a gastroscopy and an enteroclysis. No intraluminal abnormalities were observed. Colonoscopy showed only the diverticulosis.

The patient was started on prednisone 40 mg daily. Her symptoms gradually decreased in intensity and she commenced eating four days later without any nausea. She was discharged 7 days after admission with a recommendation to restart her previous anti-rheumatoid treatment. She was closely followed-up for 8 months. Methothraxate was restarted 30 days later. A CT scan was repeated 3 months later demonstrating a slight imaging improvement although not corresponding to the important clinical amelioration of the pain that totally disappeared during this period of time.

### Case 2

A 62-year-old male patient presented to us because of a large incisional abdominal wall hernia. Upon physical examination, apart from the obvious hernia, there was a sizable, mobile hard mass located at the mesogastrium and at the same level as the hernia. The patient did not mention any abdominal pain, no change in bowel habits or hematochesia and no weight loss. His past medical history was unremarkable.

**Figure 2:**
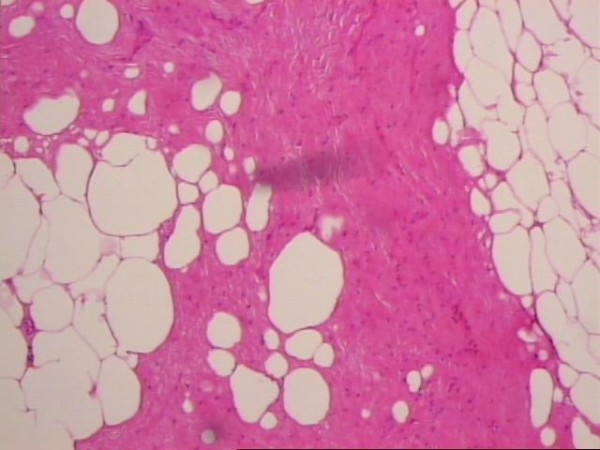
**Idiopathic retractile mesenteritis illustrating fat necrosis, sclerosing fibrosis and chronic inflammation**.

**Figure 3:**
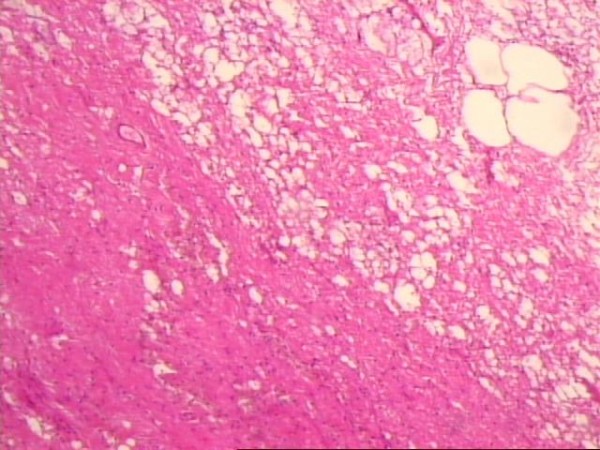
**Idiopathic retractile mesenteritis illustrating fat necrosis, sclerosing fibrosis and chronic inflammation**.

During operation, after identification of the hernia sac, just beneath the peritoneum, a sizable multinodular mass was palpated. A thick and hard mesenterium was revealed presenting with multiple yellowish nodules. Biopsies of these mesenteric nodules were obtained. Closure was performed with a biological mesh. All pathology specimens revealed fatty necrosis with fibrosis consistent with sclerosing mesenteritis but no evidence of malignancy (Figures 2, Figure 3).

**Figure 4:**
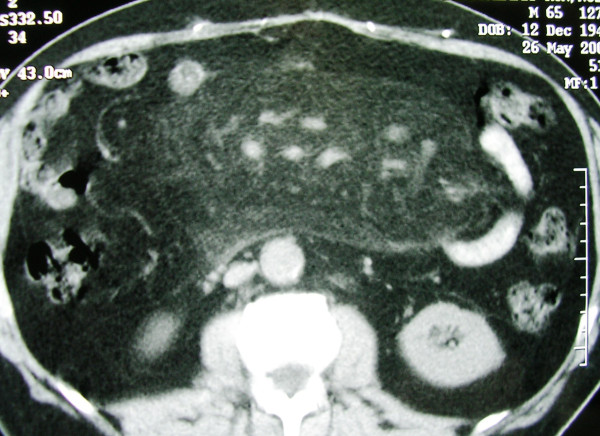
**Diffuse haziness and increased density of a thickened jejunal mesentery**. There is smooth displacement of the adjacent bowel loops. The mesenteric vessels course through the lesion without distortion.

The patient's postoperative course was uneventful. He was mobilized immediately without any special need for analgesics. He was discharged on the third postoperative day. The patient was given strict recommendation for reexamination after a month. When he came back for reevaluation, he did not report any abdominal pain or discomfort. An abdominal CT scan was performed in order to obtain a 'reference' image study for his diseases. The CT scan demonstrated characteristic findings of sclerosing mesenteritis (diffuse haziness and increased density of a thickened jejunal mesentery) (Figure 4). Due to lack of any symptoms it was not considered necessary to start any immunosuppressive medication. Additional CT scan was not proposed since the patient does not present clinical acute or chronic worsening of the disease. The patient is doing well 11 months later.

### Case 3

A 72-year-old female patient was admitted to our hospital with a three day history of recurrent abdominal pain. On clinical examination the patient appeared ill, she was febrile (38°C), in acute distress and reporting diffuse abdominal pain. There was a large movable mass palpated, extending from the pubis to the umbilicus. The patient did not report any recent weight loss and was passing flatus and stools. She had a past medical history suggestive of Dukes B sigmoid colon cancer which had been excised 36 months previously. Abdominal X-ray did not reveal any pathology, while her laboratory profile was normal.

**Figure 5:**
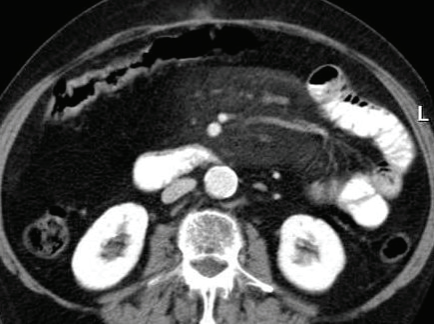
**Axial contrast enhanced CT image of the mid abdomen shows a heterogeneous fibrofatty mass within the root of the mesentery containing a focus of calcification**.

The patient had a colonoscopy which revealed no cancer recurrence or any other pathology in the remaining colon. Axial contrast enhanced CT image of the mid abdomen showed a heterogeneous fibrofatty mass within the root of the mesentery, a finding consistent with sclerosing mesenteritis (Figure 5).

Conservative treatment was commenced based on corticosteroids. The patient was started on high dose steroid therapy (methylprednisone, 80 mg iv, every 8 hours). She experienced rapid clinical improvement. She was discharged from the hospital 4 days later with a marked amelioration on a regimen of oral prednisone (40 mg/day). Prednisone was gradually interrupted after 12 weeks. Her pain gradually decreased, and 6 months later had no abdominal complaints. No additional CT scan was proposed. However, we emphasized the need of close observation of the disease in the context of her regular oncologic follow-up.

### Case 4

An 83-year-old man was admitted to our department for surgical treatment of cecal cancer that had been diagnosed elsewhere. He had a past medical history of atrial fibrillation and coronary heart disease, both on medication. He mentioned having undergone an appendicectomy in his childhood and a laparoscopic cholecystectomy three years earlier. Three months earlier, due to persistent fatigue, he had undergone a complete laboratory examination demonstrating iron deficiency anemia which was treated with oral ferum. Because of continuing weight loss, abdominal discomfort and particularly because of a non symmetric sizable palpable mass of the middle abdomen, he underwent CT of the abdomen. The CT revealed cecal thickness and radiological findings of sclerosing mesenteritis. Colonoscopy revealed a cecal mass.

**Figure 6:**
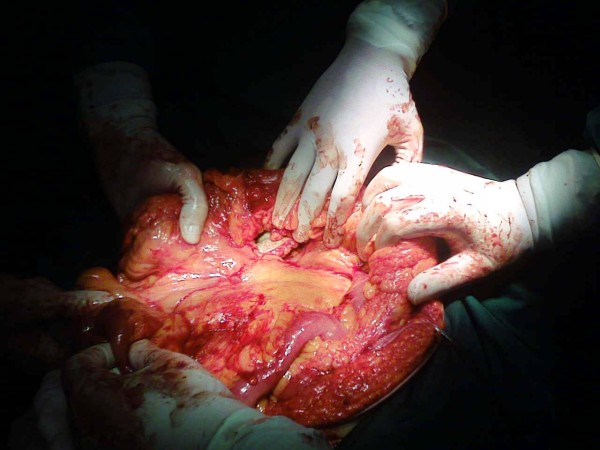
**A diffusely thick mesenterium with multiple yellowish nodules**.

At laparotomy a diffusely thick mesenterium with multiple yellowish nodules was identified (Figure 6). A right hemicolectomy was performed, and multiple biopsies of the mesenteric nodules were obtained. The pathology report described a Astler-Coller C2 cecal adenocarcinoma and diffuse sclerosing mesenteritis of the excised mesenterium. Two months later, he followed oncologic treatment consisting in capecidabine under close surveillance of his coagulation parameters. No abdominal pain or discomfort was observed during this period. CT image, performed 6 months later in the context of cancer follow up did not demonstrate important radiologic changes of the sclerosing mesenteritis.

### Case 5

A 79-year-old woman presented to the outpatient facilities because of a chronic history of recurrent abdominal pain. Her symptoms were episodic and included discomfort and nausea resulting in pain that lasted a few minutes. Since last month the pain was more intense, lasted longer periods and was relieved only with large doses of non steroid anti-inflammatory drugs. Upon physical examination she appeared well-looking, was in no acute distress and had stable vital signs. A palpable, rather painful firm abdominal mass was identified in the umbilical area.

Laboratory data revealed a normal complete blood count, blood chemistry and coagulation profile. Upper gastrointestinal endoscopy was performed and showed mild non-erosive gastritis and interspersed gastric nodules which the pathology report showed to be non malignant after they have been biopsied. She underwent an abdominal CT which showed a fatty mass within the small bowel mesentery surrounded by nodules and strictures. Such radiological findings were not indicative of a specific diagnosis, thus an exploratory laparotomy was performed in order to definitely exclude any malignancy.

**Figure 7:**
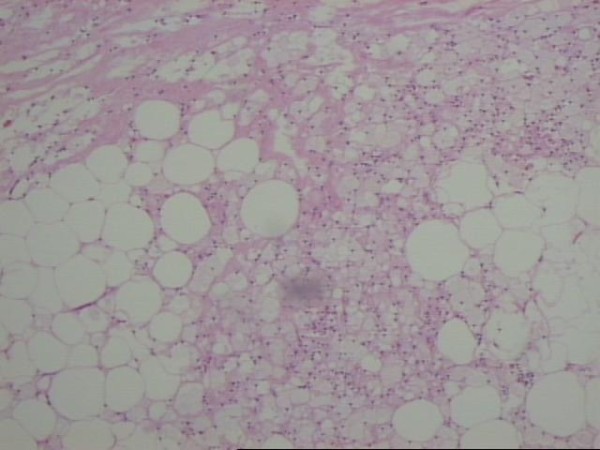
**Fatty necrosis with scarce fibrotic components, adipose cells with foamy cytoplasm and infiltration of numerous lipid-laden macrophages**.

**Figure 8:**
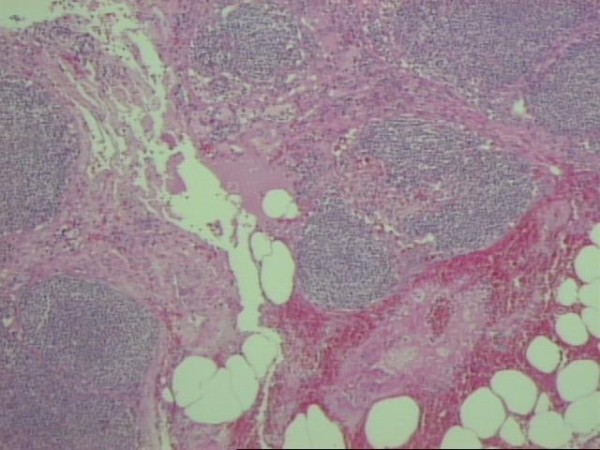
**Reactive lymphadenopathy of the mesenteric lymph nodes with expansion and alteration of the marginal zone, but without effacement of the node architecture**.

The mass was biopsied, however, a complete removal was impossible due to its encasement of the superior mesenteric vessels. The patient's postoperative course was uneventful. Pathology examination proved to be difficult because of multiple lymph nodes found in the specimen which initially presented with characters of small B-cells lymphoma. The later was excluded with polymerase chain reaction (PCR). A diagnosis of sclerosing mesenteritis was finally established (Figures 7, 8).

The patient was treated with steroids (prednisone 40 mg/daily). Clinical improvement was evident after six weeks, while four months later, the patient was symptom free. Six months later a CT was performed demonstrating an important amelioration of the radiologic appearance of the mesenteric fat.

## Discussion

Sclerosing mesenteritis is a rare disease of unknown pathogenesis represented with a various and non specific symptomatology that has become of clinical interest during recent years. Only four large series have been published in world literature, one by Durst [18], one by Kipfer [19], one by Emory [1], and the largest one more recently by Akram [10]. Other than these four series, most published reports are of single patients or small case series like ours [15,20-22], or reviews of existing literature [23-25]. Due to the rareness of published cases there is a lack of established knowledge about the exact cause, the etiological factors, the natural history, the prognosis, and finally the optimal treatment options of patients with SM.

SM is a disease of middle-aged or older adults (age range 20-90 years), primarily diagnosed during the 6^th ^to 7^th ^decade of life; the incidence increases with age, while pediatric cases are very uncommon, probably because children have less mesenteric fat than adults [10,26,27]. Most studies indicate that the disease appears to be at least twice as common in men as in women [1,18,19].

In over 90% of cases SM involves the small-bowel mesentery, but it may sometimes involve the sigmoid mesentery [28]. Exceptionally it may involve the mesocolon, peripancreatic region, omentum, retroperitoneum or pelvis [10].

The pathophysiology of SM remains unknown. The pathogenic mechanism seems to be a non-specific response to a wide variety of stimuli. It has been reported on in one series in which 84% of patients had a history of previous abdominal trauma or surgery [1]. In a recent study, Akram et al reported a history of abdominal surgery in about 40% of the patients [10]. Most of these patients had had cholecystectomy or appendicectomy between 1960 and 1990, in an era where no laparoscopic procedures had yet been developed. Before 1990 the use of powdered surgical gloves was a common practice; this fact might have a role in the formation of peritoneal adhesions and fibrosis in some cases [10,29]. Autoimmune and infective causes such as abdominal tuberculosis [9] as well as vascular insufficiency (mesenteric thrombosis, mesenteric arteriopathy, previous surgery, trauma) and retained suture material [1,11,13] have all been implicated as etiological factors. Other factors, such as coronary disease, gallstones, cirrhosis, peptic ulcer, chylous ascitis or abdominal aortic aneurysm have also been associated with this disease [27]. A recent study has shown a strong relationship between tobacco consumption and SM [13]. Additionally, SM is often associated with other idiopathic inflammatory disorders such as retroperitoneal fibrosis, sclerosing cholangitis, Riedel's thyroiditis and orbital pseudotumours [30-32].

Sclerosing mesenteritis has been associated with a variety of malignant diseases such as lymphoma, colon cancer, renal cell cancer, lung cancer, melanoma, myeloma, gastric carcinoma, Hodgkin's disease, chronic lymphocytic leukemia, thoracic mesothelioma and carcinoid tumor [1,26,13,28]. Kipfer et al. [19] found that 30% of patients with SM had an underlying malignancy. Daskalogiannaki et al. [13] reported a higher rate of coexisting malignancy (69%), most commonly urogenital malignancies and gastrointestinal adenocarcinoma or lymphoma. On the other hand, other authors have found that the prevalence of malignancy in SM is no different from the general population of patients undergoing CT for various reasons [30]. It seems that the exact pathogenetic link between SM and malignant disease is unclear. The possibility of SM representing a paraneoplastic response has been suggested [13,31].

In our series 2 out of the 5 patients (40%) had colon cancer. Nevertheless, case 3 patient had excised the sigmoid cancer 36 months prior to his admission for SM symptoms, and unfortunately we do not have any information regarding the mesenteric condition in her previous surgery. Two patients (40%) had a free medical history; thus we were not able to link their disease with any potential pathogenetic factor. One patient had a history of rheumatoid arthritis under immunosuppressant treatment with prednisone and methotrexate for several years. The abnormal immunoreactions common in autoimmune diseases could cause an inflammatory mesenteric process. The interruption of the immunosuppressive therapy probably favored the clinical presentation of the disease that had been covered during treatment.

The disease is often asymptomatic and indeed most patients in any given series were incidentally identified during a CT examination [13]. When present, clinical symptoms are non-specific and protean. The most common symptoms are abdominal pain, bloating/distension, diarrhea, nausea, vomiting, weight loss, loss of appetite, constipation and altered bowel habit [10]. Most symptoms associated with SM are caused by the direct mechanical effect of the mesenteric mass encasing the bowel, blood vessels and lymphatics, resulting in abdominal pain, bowel obstruction, ischemia and chylous ascites. Exceptionally, rectal bleeding, jaundice, gastric outlet obstruction, fever of unknown origin, autoimmune haemolytic anaemia, protein-losing enteropathy and even acute abdomen have been reported [1,10,33,34]. Upon physical examination the abdomen may be normal or there may be tenderness. Abdominal mass may be palpated in a proportion of patients that varies from study to study. Durst et al [18] report an incidence of a palpable mass of 50%, while Akram et al [10] found a significantly lower number (15%) of patients suffering from SM having a mass. Such a wide variety of signs and symptoms means that a large number of pathological conditions must be considered for differential diagnosis; lymphoma, lymphosarcoma, desmoid tumors, carcinoid tumors, peritoneal mesothelioma, amyloidosis, retroperitoneal sarcoma, infectious diseases (tuberculosis, histoplasmosis), reaction to adjacent cancer or chronic abscess, chronic inflammation due to foreign body and Whipple's disease should be part of the differential diagnosis of SM.

The diverse clinical presentation of SM was obvious in our series as well. There were two asymptomatic patients that were accidentally diagnosed as having SM during surgery for an abdominal hernia and cecal cancer respectively. The weight loss reported by one patient could not be attributed to the SM as there was an underlying malignant disease (cecal cancer). The other three patients presented with abdominal pain as the predominant symptom, while there was one patient with fever. It is remarkable that during physical examination, a palpable mass was revealed in all of the patients (100%).

Blood tests tend to be within the normal range. Neutrophilia, elevated erythrocyte sedimentation rate or anaemia have been reported occasionally in SM, but these are not specific [13,26]. While a definite diagnosis of SM requires surgical excision biopsy and pathological analysis, in the majority of cases the disease is diagnosed predominantly on the basis of CT features. In one study only 8% of patients had a biopsy proven diagnosis of SM [13]. On CT the hallmark of SM is increased density of mesenteric fat to attenuation values of -40 to -60 Hounsfield units (HU) as compared to the attenuation of normal subcutaneous and retroperitoneal fat of -100 to -160 HU [13]. CT scan appearance varies from increased attenuation ("misty mesentery") to solid soft-tissue mass, which might envelope the mesenteric vessels preserving the surrounding fatty area ("fat ring sign") [32]. The hyperattenuating fat encases the mesenteric vessels but does not displace them. Multiple masses or diffuse thickening of the mesentery are less common [3]. The mesenteric lesion may occasionally displace the adjacent small bowel loops. Calcifications associated with fat necrosis are a rare finding in this disease. In 50% of patients a tumoural pseudocapsule may be present [3,13] The small bowel mesentery is affected in most cases especially at its root; with a propensity for jejunal mesentery, but sigmoid mesocolon and omentum can occasionally be involved [30]. Exceptionally, the inflammatory process may extend into the retroperitoneum and involve the pancreas, duodenum, inferior vena cava, urinary system and pelvis [3,18]. SM may mimic imaging features of pancreatitis or even a pancreatic mass with retroperitoneal extension [6,35]. In 80% of patients with SM small soft tissue nodules can be found scattered within the mesenteric mass. These nodules are usually less than 5 mm in diameter and are thought to represent small lymph nodes [13,32]. In the study of Akram et al [10], in 61% of cases, the abdominal CT showed a single soft-tissue mass in the root of the mesentery, often containing calcification. In 34% of cases there was a subtle increase in the density of the mesenteric fat suggesting mild mesenteric fibrosis or inflammation. Small retroperitoneal and/or mesenteric lymph nodes, encasement of mesenteric vessels and collateral circulation were frequently noted. SM has also been reported to have CT findings that show similar radiological appearance to other conditions including desmoid tumors, lipoma, lymphoma, carcinoid tumors, liposarcoma, tuberculosis, mesothelioma, mesenteric metastases, edema or hematoma [36,37].

A part from CT, magnetic resonance imaging (MRI) seems to be helpful in the detection of SM [38]. PET-CT has been proposed to have a promising role in differentiating between benign SM and co-existing SM and mesenteric tumoural involvement particular in patients with lymphoma [8].

In our cases, four patients underwent a CT scan in an effort to establish a diagnosis. CT was a valuable tool in excluding abdominal pathology that might need urgent surgical intervention. In addition, in two cases it was the CT findings that suggested the conservative/medical treatment as the therapeutic option of choice. Nevertheless CT findings cannot distinguish the severity of the disease.

Histologically, sclerosing mesenteritis displays different stages of involvement [26]. The first stage involves mesenteric lipodystrophy in which a layer of foamy macrophages replaces the mesenteric fat. Signs of acute inflammation are absent or minimal; the disease tends to be clinically asymptomatic and prognosis is good. In the second stage, termed mesenteric panniculitis histology reveals an infiltrate made up of plasma cells and a few polymorphonuclear leukocytes, foreign-body giant cells and foamy macrophages. Most common symptoms include fever, abdominal pain and malaise. The final stage is retractile mesenteritis which is distinguished by collagen deposition and a diffuse presence of necrosis and fibrosis that contribute to tissue retraction. Collagen deposition leads to scarring and retraction of the mesentery which in turn leads to the formation of abdominal masses and obstructive symptoms. The exact diagnosis is often difficult and is usually made by finding one of three major pathological features; fibrosis, chronic inflammation or fatty infiltration of the mesentery. To some extent all three components are present in most cases. This pathological lesion involves the mesenteric and submucosal fat of the small bowel often with extension into the bowel muscle and submucosa. However the mucosa usually remains intact [32].

A large series by Kipfer et al [19], and Akram et al [10] reported that 74% and 50% respectively of patients underwent surgical exploration in order to establish a diagnosis. With better non-invasive/imaging diagnostic methods fewer patients require exploratory surgery unless indicated by intractable complications of SM (small bowel obstruction, superior mesenteric venous thrombosis, lower gastrointestinal bleeding), or if there is high clinical suspicion of an alternative diagnosis. However attempted surgical resection or debulking usually does not result in resolution of the symptoms or prevent disease progression as evidenced by only 10% of our patients improving after surgery alone [10].

There is no consensus of opinion on medical treatment for symptomatic cases of SM. A variety of anti-inflammatory, immunomodulatory, and antifibrotic agents are used. Drug therapy is not standardized and should be based on the stage of the disease. In the first stage (lipodystrophy) when fat necrosis is predominant, authors agree not to treat the disease as it can regress spontaneously. Chronic inflammation requires therapy based on corticosteroids and various types of immunosuppressants. Good results are reported on with cyclophosphamide, colchicine, azathioprine, thalidomide, and also with oral progesterone and tamoxifen [10,11,17,34,39,40]. Pentoxyfylline has been recently reported as promising antifibrotic agent successfully used in a case of sclerosing mesenteritis [41]. Tamoxifen was successfully used in 19 patients in the series of Akram et al. [10] and 63% of these patients responded within 12 weeks of initiation of treatment. In the same series no similar benefits were obtained with prednisone alone or in combination with non-tamoxifen treatment. In contrast, clinical improvement was observed with oral corticosteroid treatment alone in two patients of our small series. As intense fibrosis appears bowel obstruction may occur. Intestinal resections, bypasses or neostomy might be required.

In the first case of our series the combined use of corticosteroids and methotrexate against rheumatoid arthritis probably maintained SM in a tolerant state. When this balance was interrupted, the disease brought out its clinical manifestations. As soon as the patient restarted the immunosuppressive treatment for rheumatoid arthritis she had a marked clinical improvement. Although, it is not clear if the clinical benefit was due to cortisone, methothrexat or a synergic effect of both. In case 2, treatment was not administered because there were no specific symptoms. In case 4, additional oncologic treatment was added and one could await an anti-proliferative and immunosupressive effect. However, no particular radiologic changes were observed. In case 3 and 5, conservative treatment was commenced based on corticosteroids. The patients experienced rapid clinical improvement. In these cases steroid therapy gave very satisfactory results, therefore it was not necessary to use any other medications. We should also emphasize that CT scan was repeated in three cases (case 1, 4, 5). Particular amelioration of the radiologic findings was observed only in one case. However, all patient presented important clinical improvement. This fact is in contrast with other reported cases where therapy was followed by a radiological improvement [12]. Akram et al [10] consider that the management of the disease should be based on severity and type of symptoms and not on CT findings. The author does not recommend serial CT follow-up, unless clinical deterioration is observed. Our data further support this opinion.

It seems that SM has different modes of progression, a complete or partial resolution, a non progressive course with stable clinical symptoms that can be controlled with drugs and a progressive course that may be fatal [39,40]. Nevertheless, the disorder has generally been reported as having a self- limiting benign course with spontaneous remissions being the most favourable outcome. Although the overall prognosis is good, in about 20% of patients, SM is associated with significant morbidity and a chronic debilitating course [16].

## Conclusions

Sclerosing mesenteritis is a rare idiopathic disorder that involves predominantly the small bowel mesentery with varying degrees of fibrosis, inflammation and fat necrosis. Diagnosis of this nonspecific benign inflammatory disease is a challenge to surgeons, radiologists, gastroenterologists and pathologists. Its clinical presentation is quite diverse and ranges from being asymptomatic to a debilitating disease. CT features of the disease, usually highly suggestive, have recently been delineated clearly. Approximately half of the patients may not require any treatment. However, in symptomatic cases treatment should be tailored according to the severity and type of individual symptoms. Patients with SM related complications like intractable bowel obstruction should undergo surgery, while those with nonobstructive symptoms might benefit from steroid therapy alone or in combination with other drugs. Overall prognosis is usually good and recurrence seems to be rare. However long-term follow-up is needed to document these results.

## Consent

Written informed consent was obtained from all patients for publication of their medical data.

## Competing interests

The authors declare that they have no competing interests.

## Authors' contributions

KVL, EF and SM participated to the sequence alignment, researched sources for the reference and drafted the manuscript. KVL, SK took the photographs and drafted the manuscript. FA, CV helped in the interpretation of the photos and helped draft the final version of the manuscript. All authors read and approved the final manuscript form.
